# Supplementary material for: A Pangenomic Approach to Improve Population Genetics Analysis and Reference Bias in Underrepresented Middle Eastern and Horn of Africa Populations
Source: Biomolecules. 2025 Apr 15;15(4):582. doi: 10.3390/biom15040582 (PMC12025191; doi:10.3390/biom15040582)
Supplement: Supplementary file 1 [file biomolecules-15-00582-s001.zip › biomolecules-3512081-supplementary.pdf]

# Supplementary Information for

## A Pangenomic Approach to Improve Population Genetics Analysis and Reference Bias in Underrepresented Middle Eastern and Horn of Africa Populations

**Adrien Oliva [1,*], Rachel Foare [2], Peter Campbell [3], Natalie A. Twine [1], Denis C. Bauer [1] and Angad Singh Johar [4]**

[1] Australian e-Health Research Centre, Commonwealth Scientific and Industrial Research Organisation (CSIRO), Victoria 3169, Australia; natalie.twine@csiro.au (N.A.T.); denis.bauer@csiro.au (D.C.B.)
[2] Life Sciences and Health Graduate School, Université Paris-Saclay, 3 Rue Joliot Curie, 91190 Gif-sur-Yvette, France; rachel.foare@gmail.com
[3] Information Management and Technology (IM&T), Commonwealth Scientific and Industrial Research
Organisation (CSIRO), Victoria 3169, Australia; peter.h.campbell@csiro.au
[4] Menzies Institute of Medical Research, The University of Tasmania, Hobart 7000, Australia; angad.johar@utas.edu.au
* Correspondence: adrien.oliva@csiro.au

**This PDF file includes:**

- Supplementary Table S1 and captions.
- Supplementary Figures S1, S2 and captions.
- Supplementary Text.

# Table of Contents

**Supplementary Table** ........ 3

**Supplementary Figures** ........ 6

**Supplementary Text** ........ 8

**References** ........ 10

# Supplementary Table

| Population Pairs | $F_{ST}$ Value |
|---|---|
| Somali vs Ethiopian Jews (Ethiopian_BetaIsreali) | 0.0103 |
| Somali vs Mozabite | 0.0430 |
| Somali vs LWK (Luhya West Kenya) | 0.0509 |
| Somali vs YRI (Yoruba Ibadan, Nigeria) | 0.0574 |
| Somali vs MSL (Mende in Sierra Leone | 0.0507 |
| Somali vs ESN (Esan in Nigeria) | 0.0510 |
| Somali vs GWD | 0.0587 |
| Ethiopian Jews vs Mozabite | 0.0293 |
| Ethiopian Jews vs LWK | 0.0512 |
| Ethiopian Jews vs YRI | 0.0628 |
| Ethiopian Jews vs MSL | 0.0624 |
| Ethiopian Jews vs ESN | 0.0631 |
| Ethiopian Jews vs GWD | 0.0598 |
| Mozabite vs LWK | 0.0954 |
| Mozabite vs YRI | 0.103 |
| Mozabite vs MSL | 0.104 |
| Mozabite vs ESN | 0.1050 |
| Mozabite vs GWD | 0.101 |
| LWK vs YRI | 0.0075 |
| LWK vs MSL | 0.0102 |
| LWK vs ESN | 0.0083 |
| LWK vs GWD | 0.0111 |
| YRI vs MSL | 0.0041 |
| YRI vs ESN | 0.0011 |
| YRI vs GWD | 0.0063 |
| MSL vs ESN | 0.0054 |

| | |
|---|---|
| MSL vs GWD | 0.0039 |
| GWD vs ESN | 0.0078 |
| Somali vs Palestinian | 0.0524 |
| Somali vs Bedouin | 0.0496 |
| Somali vs Druze | 0.0638 |
| Mozabite vs Palestinian | 0.0231 |
| Mozabite vs Bedouin | 0.0230 |
| Mozabite vs Druze | 0.0288 |
| Palestinian vs Bedouin | 0.00810 |
| Palestinian vs Druze | 0.00935 |
| Bedouin vs Druze | 0.0121 |
| Ethiopian Jews vs Bedouin | 0.0468 |
| Ethiopian Jews vs Palestinian | 0.0341 |
| Ethiopian Jews vs Druze | 0.0438 |
| Druze vs LWK | 0.131 |

| | |
|---|---|
| Druze vs ESN | 0.142 |
| Druze vs GWD | 0.138 |
| Druze vs MSL | 0.144 |
| Druze vs YRI | 0.145 |
| Palestinian vs LWK | 0.116 |
| Palestinian vs ESN | 0.129 |
| Palestinian vs GWD | 0.125 |
| Palestinian vs MSL | 0.128 |
| Palestinian vs YRI | 0.129 |
| Bedouin vs LWK | 0.115 |
| Bedouin vs ESN | 0.127 |
| Bedouin vs GWD | 0.122 |
| Bedouin vs MSL | 0.126 |
| Bedouin vs YRI | 0.125 |

**Table S1:** Pairwise $F_{ST}$ between Africans and non-Africans analysed in the study. The distances are calculated using the Weir-Cockerham algorithm as implemented in *ADMIXTOOLS2*. The dataset includes all African and Middle Eastern populations from Human Origins, HGDP, and

1000 Genomes Phase 3. Distances are indicators for populations with the most proximity to the HOA population of interest, an essential first step for graph building in vg.
Abbreviated Population names are as follows: ‘LWK’ = Luhya in West Kenya, MSL = Mende in Sierra Leone, ESN = Esan in Nigeria, GWD = Gambia Western Division, YRI = Yoruba in Ibadan, Bedouin = Negev Beoduin populations from the HGDP

# Supplementary Figures

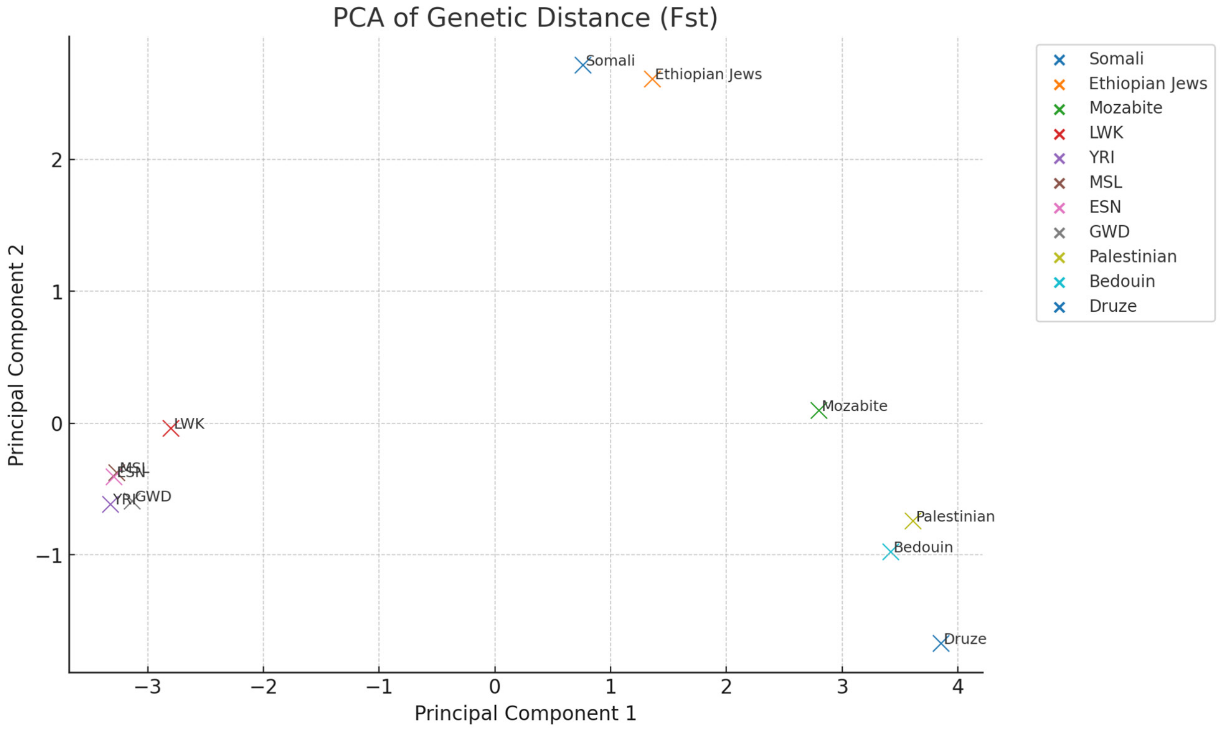


**Figure S1:** Principal Component Analysis (PCA) performed using all pairwise *Fst* distances from Table S1. The first two Principal Components (PCs) summarise the largest variance within the multidimensional *Fst* space, providing insight into population structure and genetic relationships. The total variance explained by these two PCs is reported.

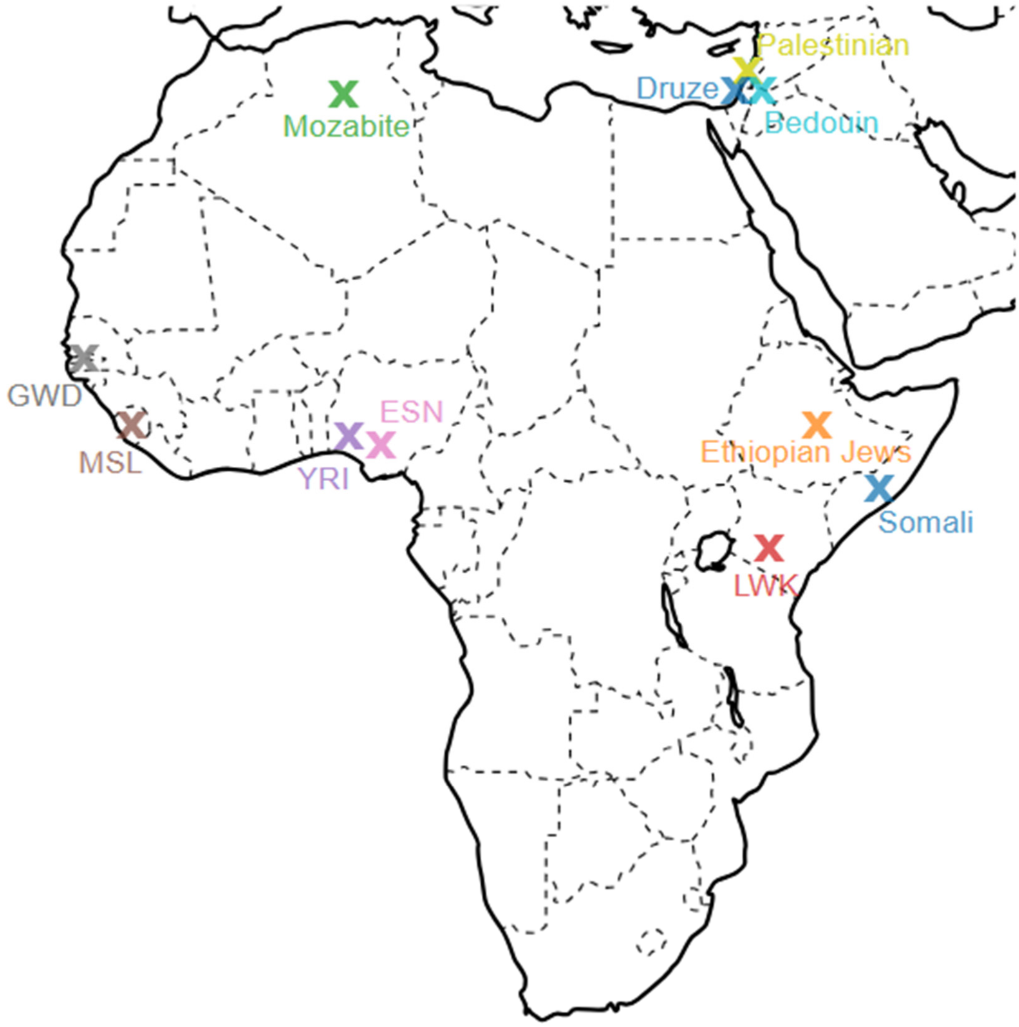


**Figure S2:** Geographic distribution of samples used in this study. The populations are sourced from HGDP, 1000 Genomes Phase 3, and Human Origins datasets. These sample locations provide demographic context and help visualize the relationship between geography and genetic structure across Africa and neighbouring populations.

Abbreviated Population names are as follows: 'LWK' = Luhya in West Kenya, MSL = Mende in Sierra Leone, ESN = Esan in Nigeria, GWD = Gambia Western Division, YRI = Yoruba in Ibadan, Bedouin = Negev Bedouin populations from the HGDP

# Supplementary Text

A key component of this study is the comparison of Somali genetic diversity with available African and Middle Eastern populations. To achieve this, we conducted $F_{ST}$ analyses using the *ADMIXTOOLS2* algorithm [1] on selected populations (Table S1) from the Allen Ancient DNA Resource (AADR) dataset [2], followed by a Principal Component Analysis (PCA) [3]. In total 593K SNPs (lifted from hg19 to hg38) from the AADR were used [2,4]. Our results reveal that Somalis occupy an outlier position in PCA space, clustering only with Ethiopian Jews (Beta Israel), while remaining distinct from 1000 Genomes Africans and HGDP Mozabites (Supplementary Figure S1, Table 1). This pattern aligns with findings from Ali et al. (2020) [5] and Koenig et al. (2024) [6] reinforcing the unique genetic profile of Somalis and other Horn of Africa (HOA) populations, such as Oromo Ethiopians. In Ali et al. [5], HOA populations—particularly Somalis—form distinct clusters in PCA space compared to other African groups. Notably, Ethiopian Jews exhibit the lowest $F_{ST}$ differentiation with Somalis, which may explain why they, along with Iron Age Kenyans, emerge as key source populations for Somalis in *qpAdm*, despite their cultural differences. This observation is consistent with Hodgson et al. [7], who describe how Indigenous African ancestry in the HOA is largely restricted to Ethiopian and Somali groups. Additionally, their non-African genetic component, known as the Ethio-Somali composition, is thought to have originated from ancient Back-to-Africa migrations and remains genetically distinct from Northern Maghrebi groups. Interestingly, Sudanese populations do not exhibit the same level of genetic proximity to Somalis as Ethiopian Jews [7]. This suggests that genetic diversity patterns across Africa are shaped not only by geographic factors but also by complex historical admixture and genetic drift.

While our findings support the development and evaluation of a pangenome graph for underrepresented Horn of Africa (HOA) populations, particularly Somalis, several additional factors must be considered. Our approach, guided by PCA, external literature, *qpAdm* [1,8] , and $F_{ST}$ analyses, highlights key challenges. One major limitation is the scarcity of whole-genome data for Somali and Ethiopian HOA populations. Additionally, as previously noted, the genetic ancestry of modern Somalis has been significantly influenced by non-Indigenous contributions, especially from Middle Eastern and North African (MENA) populations [5]. To account for this, we performed extensive $F_{ST}$ and *qpAdm* analyses, incorporating prior research, and included Mozabites and other Middle Eastern groups in our *qpAdm* models. Although Mozabites (HGDP) have a higher $F_{ST}$ with Somalis than Ethiopian Jews, their $F_{ST}$ values with Somalis remain lower than those observed for West African (MSL, LWK, ESN) and East African (LWK) populations. At the same time, Mozabites exhibit lower $F_{ST}$ values with Bedouins, Druze, and Palestinians compared to the Human Origins Array Somalis. This pattern aligns with findings from

Hodgson et al. [7], which analyzed $F_{ST}$ relationships between Middle Eastern (Palestinian, Bedouin, Druze), Maghrebi, and Ethio-Somali populations.

The most parsimonious theory explaining genetic relationships between the Maghrebi and Horn of Africa (HOA) populations is supported by multiple lines of evidence, including $F_{ST}$, ADMIXTURE, coalescence of uniparental haplogroups, and hierarchical population tree models based on ancestry partitioning and AMOVA analyses. This model, proposed by Hodgson et al. [7].suggests that both regions share ancestry from a single Back-to-Africa migration, with their lineages later diverging and experiencing separate, more recent gene flow from West Asia [7]. Further supporting this, genetic differentiation analyses ($F_{ST}$ and AMOVA) indicate that HOA populations are significantly more differentiated than those in the Maghreb, suggesting that genetic drift has had more time to act in the HOA. While there is some evidence of gene flow from the Maghreb into the HOA, both regions exhibit substantial substructure, and the non-African ancestry in the HOA cannot be fully explained by Maghrebi gene flow alone.

Building on these findings, Koenig et al. [6] show that Mozabites are clearly separated from Palestinians, Bedouins, and Druze in PCA space when considering the first two Principal Components (PCs). However, on the third and fourth PCs, there is considerable overlap between HGDP Mozabites and HGDP Bedouins, indicating a degree of shared genetic diversity.

Additionally, our $F_{ST}$ -based PCA (Figure S1) positions Mozabites between the three Middle Eastern populations and the HOA, aligning with previous findings on HOA and Mozabite genetic differentiation from other African populations [7,9–11]. In combination with our *qpAdm* [1,8] and $F_{ST}$ results, these patterns suggest that Mozabites are the best available proxy for representing the non-African, West Asian genetic component in HOA populations. Despite the substructure between MENA and HOA, both our findings and previous studies indicate that HGDP Mozabites, likely due to shared ancestry from Back-to-Africa migrations, are the most suitable reference population for variation graph construction, while HGDP Bedouins are optimal for evaluation.

# References


1. Maier, R.; Flegontov, P.; Flegontova, O.; Işıldak, U.; Changmai, P.; Reich, D. On the Limits of Fitting Complex Models of Population History to -Statistics. *Elife* **2023**, *12*, doi:10.7554/eLife.85492.
2. Mallick, S.; Micco, A.; Mah, M.; Ringbauer, H.; Lazaridis, I.; Olalde, I.; Patterson, N.; Reich, D. The Allen Ancient DNA Resource (AADR) a Curated Compendium of Ancient Human Genomes. *Scientific Data* **2024**, *11*, 1–10.
3. Novembre, J.; Stephens, M. Interpreting Principal Component Analyses of Spatial Population Genetic Variation. *Nature genetics* **2008**, *40*, doi:10.1038/ng.139.
4. Perez, G.; Barber, G.P.; Benet-Pages, A.; Casper, J.; Clawson, H.; Diekhans, M.; Fischer, C.; Gonzalez, J.N.; Hinrichs, A.S.; Lee, C.M.; et al. The UCSC Genome Browser Database: 2025 Update. *Nucleic acids research* **2025**, *53*, doi:10.1093/nar/gkae974.
5. Ali, A.A.; Aalto, M.; Jonasson, J.; Osman, A. Genome-Wide Analyses Disclose the Distinctive HLA Architecture and the Pharmacogenetic Landscape of the Somali Population. *Sci Rep* **2020**, *10*, 5652.
6. Koenig, Z.; Yohannes, M.T.; Nkambule, L.L.; Zhao, X.; Goodrich, J.K.; Kim, H.A.; Wilson, M.W.; Tiao, G.; Hao, S.P.; Sahakian, N.; et al. A Harmonized Public Resource of Deeply Sequenced Diverse Human Genomes. *Genome Res* **2024**, *34*, 796–809.
7. Hodgson, J.A.; Mulligan, C.J.; Al-Meeri, A.; Raaum, R.L. Early Back-to-Africa Migration into the Horn of Africa. *PLoS Genet* **2014**, *10*, e1004393.
8. Harney, É.; Patterson, N.; Reich, D.; Wakeley, J. Assessing the Performance of qpAdm: A Statistical Tool for Studying Population Admixture. *Genetics* **2021**, *217*, doi:10.1093/genetics/iyaa045.
9. Lucas-Sánchez, M.; Abdeli, A.; Bekada, A.; Calafell, F.; Benhassine, T.; Comas, D. The Impact of Recent Demography on Functional Genetic Variation in North African Human Groups. *Mol Biol Evol* **2024**, *41*, doi:10.1093/molbev/msad283.
10. Vilà-Valls, L.; Abdeli, A.; Lucas-Sánchez, M.; Bekada, A.; Calafell, F.; Benhassine, T.; Comas, D. Understanding the Genomic Heterogeneity of North African Imazighen: From Broad to Microgeographical Perspectives. *Sci Rep* **2024**, *14*, 9979.
11. Serradell, J.M.; Lorenzo-Salazar, J.M.; Flores, C.; Lao, O.; Comas, D. Modelling the Demographic History of Human North African Genomes Points to a Recent Soft Split Divergence between Populations. *Genome Biol* **2024**, *25*, 201.
